# Supplementary material for: Integrated Transcriptome and Proteome Analysis Reveals That Cell Wall Activity Affects Phelipanche aegyptiaca Parasitism
Source: Plants (Basel). 2024 Mar 18;13(6):869. doi: 10.3390/plants13060869 (PMC10974318; doi:10.3390/plants13060869)
Supplement: Supplementary file 1 [file plants-13-00869-s001.zip › Supplementary Table S1.pdf]

Table S1 qRT-PCR primer of selected gene in transcriptome

Gene	Primer	Forward and reverse primers 5’ – 3’
Patublin1	Sense	GGTCCCGAAAGATGTCAACGC
	Anti-sense	GAGAACACCTCCGCCACGCT
TR301_c1_g1	Sense	ACTGGCTTCAACGACACCAA
	Anti-sense	ACTCTCTCCCTGTTTCCCGA
TR2875_c2_g2	Sense	ACACTACTGCGCCGAGAATC
	Anti-sense	ATTCGCCTCGAGTCTGCTTC
TR10437_c0_g1	Sense	CCCTTGGAGCAGGTGCAATA
	Anti-sense	ACCAGCAAACCACAGTGTCA
TR12713_c0_g1	Sense	AAGCCTACTAATCGCCTCGC
	Anti-sense	TTCACCTTCGACGACGCTAC
TR14230_c0_g1	Sense	TCCCACCACCTCCACCAATA
	Anti-sense	GGAGTCCGTTTGGTGTGTCT
TR20167_c0_g1	Sense	TTCAGTGTTCTACCCGTGCC
	Anti-sense	CCGGAAAACCACAAACGACC
TR53357_c0_g1	Sense	AGGTCTACTAGTGGAGGCCG
	Anti-sense	TCATGGCCTGCTCATTCACA
TR65907_c0_g1	Sense	GGAACGAGGAGCAAAATGCG
	Anti-sense	AGCAGCTATGGGGAAGGAGA
TR13481_c0_g1	Sense	ATCCTTCAGACCGCCTCTCT
	Anti-sense	GACGGAGGGGACGATGATTC
