# Supplementary figures and images for: Integrated Transcriptome and Proteome Analysis Reveals That Cell Wall Activity Affects Phelipanche aegyptiaca Parasitism
Source: Plants (Basel). 2024 Mar 18;13(6):869. doi: 10.3390/plants13060869 (PMC10974318; doi:10.3390/plants13060869)

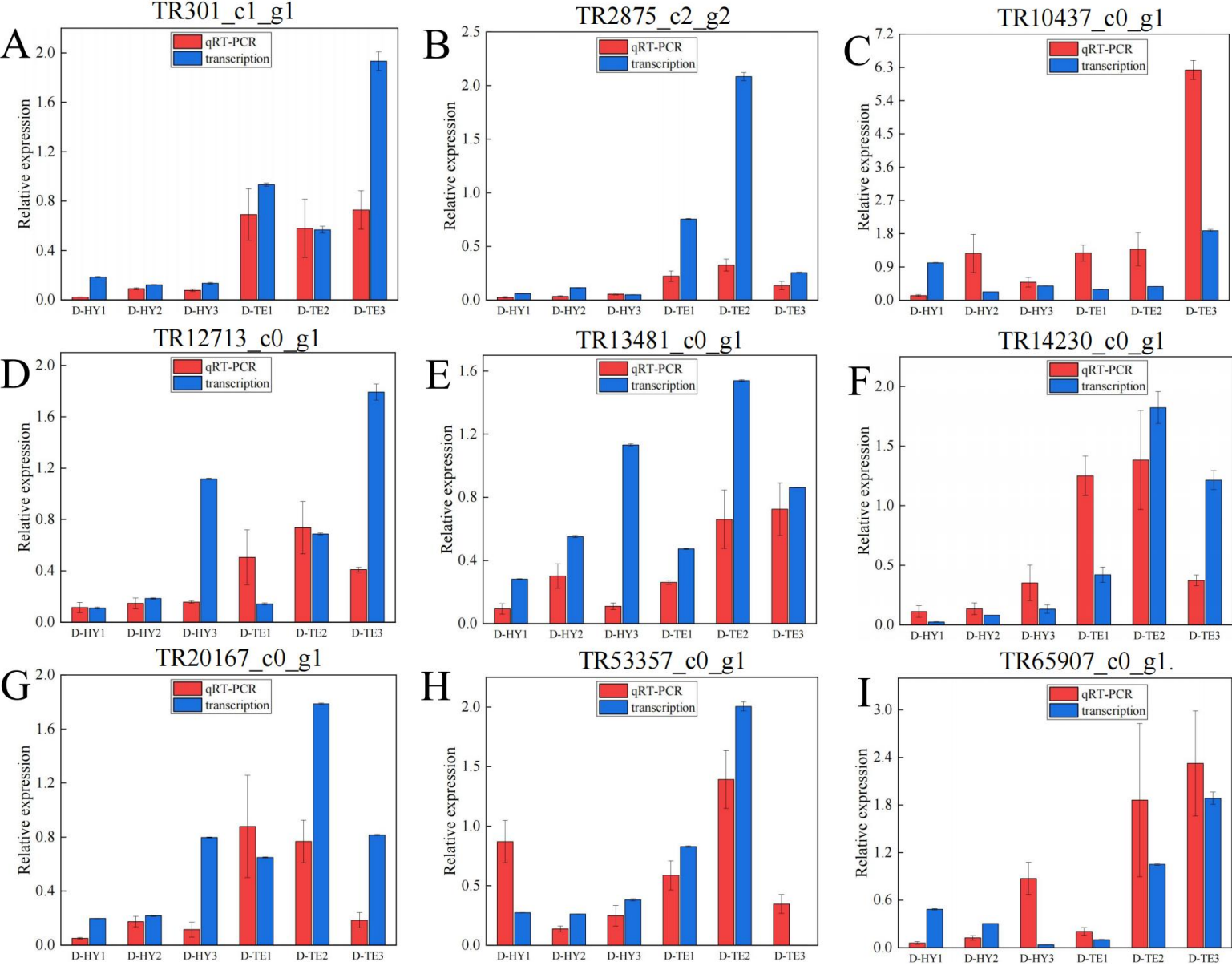

**Figure S1.** The qRT-PCR results of nine selected genes in transcriptome

Supplement: Supplementary file 1 [file plants-13-00869-s001.zip › Supplementary Figure S1.pdf]
